# Evaluation of autonomic cardiac modulation and body composition in elderly submitted to the protocol of exercise and the gametherapy: A clinical trial protocol (SPIRIT compliant)

**DOI:** 10.1097/MD.0000000000031236

**Published:** 2022-11-25

**Authors:** Sáskia Fürstenberg Thoma, Andrés Ricardo Pérez-Riera, Eduardo Antônio Costa Silva, Ester Laura Cordeiro Oliveira Costa, Airlon Nery Ferreira, Emanuel Ewald Thoma Belfort, Jéssica Costa Leite, Juliana Zangirolami-Raimundo, Rodrigo Daminello Raimundo

**Affiliations:** a Department of Physical Therapy, Centro Universitário UNIFACISA, Itararé, Campina Grande, Paraíba, Brazil; b Study Design and Scientific Writing Laboratory, Centro Universitário FMABC, Santo André, São Paulo, Brazil.

**Keywords:** aged, autonomic nervous system, experimental games, gametherapy, heart rate variability

## Abstract

Aging affects the cardiovascular system by impairing the amount and strength of cardiac pumping. Heart rate variability (HRV) is a safe parameter to assess variations in the cardiovascular system, providing data on sympathetic and parasympathetic activations.

**Objectives:**

Our primary aim is to investigate the cardiac autonomic modulation and body composition of active older adults participating in a physical exercise protocol and gametherapy. Our secondary aim is to assess their functional capacity, cognitive function, balance, respiratory pressures, and functional autonomy.

**Method:**

This randomized clinical trial will include 100 active older adults aged 60 to 80 years. The exercise group (EG) will perform 24 supervised training sessions (strength and aerobic) for 12 weeks (2 60-minutes sessions per week). The gametherapy group (GG) will exercise using gametherapy. Assessments will occur on the first week, after the 12th week, after wash out and in the end of cross over. The primary outcome will be HRV and body composition (bioimpedance). Secondary outcomes will be functional capacity (6-minute walk test), cognitive function (mini-mental state examination), risk of falls and balance (berg balance scale and timed up and go test), inspiratory and expiratory pressures (manovacuometry) and functional autonomy (functional reach test and group of Latin American development to maturity [GDLAM] protocol).

**Discussion:**

This study will provide relevant information about the effects of physical training (physical exercises and gametherapy) on HRV and other variables in active older adults.

## 1. Introduction

Population aging is a worldwide phenomenon, more than 14% of the population ages over 65 years in most European countries. The concept of successful or active aging first appeared in the 1960s and was associated with physical well-being and satisfaction with life. For a positive aging experience, older adults must actively participate in society, engage in interpersonal relationships, and maintain cognitive function, mental, and physical health. Regular physical exercise and healthy eating habits are crucial to improving cognitive, metabolic, bodily, and cardiac functions in older adults and minimizing aging effects.^[1]^

According to Hong et al,^[2]^ aging is an involuntary and inevitable process that causes progressive structural and functional decrease in body functions, functional capacity, muscle and bone mass, strength, hormones, and cardiovascular function (e.g., decrease heart rate). These losses in function may lead to disabilities, injuries, multiple illnesses, hospitalizations, falls, and mortality.^[3]^ Rezende et al^[4]^ highlight the relationship between advanced age, compromised longevity, decreased muscle strength, and overweight, it’s can be associated a change body composition, induce sarcopenia and fat accumulation, and increase the prevalence of chronic diseases and functional disabilities.

Bioimpedance is a validated instrument to assess body composition (i.e., fat percentage, muscle mass, and bone age). Bioimpedance data can indicate and help to predict chronic degenerative diseases, this instrument became popular over the years due to its low cost and practicality.^[4,5]^ Regarding the cardiovascular system, heart rate and cardiac autonomic activity may decrease with aging. Rajendra et al^[6]^ state that the sympathetic and parasympathetic nervous systems regulate the heart rate, adapting it to different conditions. Increase in heart rate is associated with greater sympathetic than parasympathetic activity. Heart rate variability (HRV) is a safe parameter to analyze autonomic activations responsible for controlling heart rate in multiple physiological and environmental stimuli.^[7]^

Given functional decline associated with aging, more knowledge is needed on the relationship between cardiac autonomic modulation and body composition of older adults submitted to an aerobic and strength exercise and gametherapy.

## 2. Material and Methods

### 2.1. Study design and protocol registration

This is a parallel-group randomized clinical trial registered at REBEC (Clinical Trial registration # RBR-7y24t7t). The research protocol was reviewed and approved by the research ethics committee of Centro de Ensino Superior e Desenvolvimento da Paraíba (#4462499 - CAAE: 39751620.0.0000.5175).

### 2.2. Recruitment and eligibility criteria

#### 2.2.1. Recruitment.

**Figure 1.**
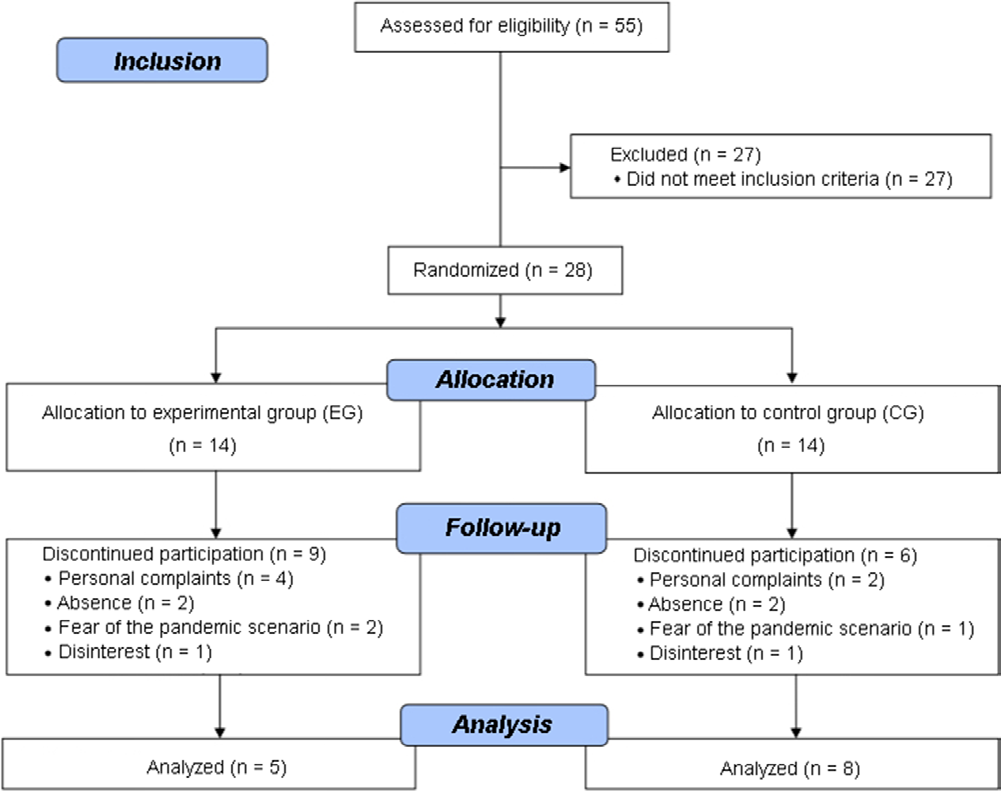
Flowchart of participant selection process based on the 2010 consolidated standards of reporting trials (CONSORT).

Participants will be recruited by disclosing the study on social media and rehabilitation clinics in Campina Grande and near cities. Potential participants will be contacted by phone to ensure that they meet inclusion criteria. The sample will consist of 100 older adults who meet the eligibility criteria. They will be randomly divided into 2 groups. Figure 1 represents the recruitment process based on the 2010 consolidated standards of reporting trials.

#### 2.2.2. Eligibility criteria.

Inclusion criteria:

- Physically “active” or “high active” older adults according to the International Physical Activity Questionnaire short form.
- People of both sexes aged from 60 to 80 years living in Campina Grande or adjacent cities.
- Older adults with diagnosis of chronic degenerative diseases under controlled medication will also be included.

Exclusion criteria:

- Older adults with pathologies impairing the performance of aerobic activities.
- Older adults with musculoskeletal disorders impairing participation in the intervention sessions.
- Older adults who use walking devices.

### 2.3. Sample size

From the 4 factors needed to calculate sample size (i.e., significance level, power, difference between groups, and standard deviation), 2 are unknown (difference between groups and standard deviation) because no previous study used HRV for older adults. Although pilot randomized controlled trials do not require sample size calculations, 15 to 20 participants per group would be necessary to ensure validity. Therefore, we will include 100 participants, 50 participants per group. Our results will provide the information necessary for sample size calculation in future studies.

### 2.4. Randomization

Participants will be randomly assigned to the exercise group (EG) (physical exercise protocol; EG) or the gametherapy group (GG) (gametherapy; GG). Simple randomization sequence will be obtained from randomization.com. Individual allocations will be sealed in an opaque and numbered envelope in sequential order in which participants will be drawn.

Group allocation will be revealed after we confirm that participant meets inclusion criteria, signs the consent form, and completes the initial assessment. All study-related information will be securely stored, and participants will be identified with a code to protect their identity. Records with names or other personal identifiers (i.e., address and informed consent forms) will be identified with a code, stored separately from study records, and accessible only by the research team.

### 2.5. Blinding

Due to the nature of the intervention, participants will be aware of the group they will be allocated to. However, researchers assessing primary and secondary outcomes will be blinded. Participants will be instructed not to disclose their allocation to the study team. Researchers analyzing the data will also be blinded.

### 2.6. Data collection

#### 2.6.1. Assessments plan.

**Figure 2.**
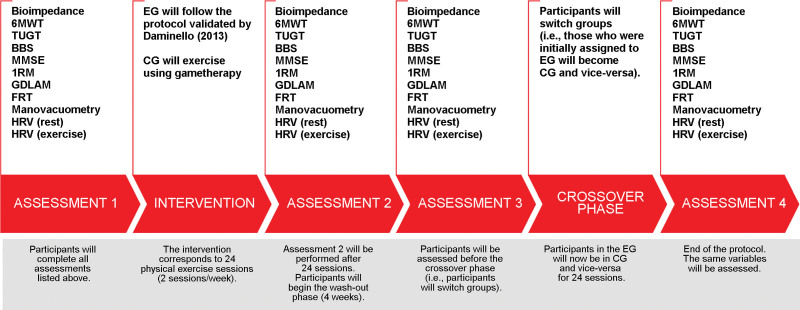
Data collection plan. 1RM = 1-repetition maximum test, 6MWT = 6-minute Walk Test, BBS = berg balance scale, CG = control group, EG = experimental group, FRT = functional reach test, GDLAM = Latin American group for maturity, HRV = heart rate variability, MMSE = mini-mental state examination, TUGT = timed up and go test.

Figure 2 represents the assessment data collection plan. Participants will be assessed in 4 moments: Assessment 1 at the beginning of the protocol, Assessment 2 after 24 sessions, Assessment 3 at the end of the wash-out phase (no intervention for 4 weeks), and Assessment 4 after 24 sessions (crossover phase or switch between protocols).

#### 2.6.2. Data collection related to the primary aim (HRV) and (body composition).

HRV will be assessed at rest and during exercise using the Polar V800. The Polar V800 is a heart rate monitor that captures electrical impulses from the heart. A device similar to a watch will be placed on participants’ left wrist, and a chest strap with a capture sensor will be positioned on their xiphoid process.

The assessment at rest will describe participants’ cardiac autonomic profile. Participants will be quiet and rest in supine position for 20 minutes in an air-conditioned room (22°C–25°C).

The assessment during exercise will be completed in 2 moments: pre- and post-intervention. The cardiac responses to the different phases of the exercise (i.e., 5-minutes warm-up, 30-minutes aerobic training, 20-minutes strength training of peripheral muscles, and 5-minutes relaxation) will be observed. EG will complete a physical exercise protocol validated by Raimundo et al,^[8]^ and GG will perform gametherapy (Just Dance 2013) Marques et al.^[9]^

HRV will be measured at rest and during exercise using linear methods in the time and frequency domains, non-linear methods, and Poincaré graph. Data analysis will be based on the heart rate recorded during the protocol, and only time series with more than 95% of sinus beats will be used for analysis. After checking for errors, a trace will be selected for analysis and filtered using the Polar Precision Performance SW software (version 4.01.029). Analysis will be performed using the Kubios software.^[9]^

Body composition will be assessed using electrical bioimpedance. Participants will be fasting for 2 hours, with empty bladder (used the bathroom 30 minutes before), and will not have practiced physical activity in the previous 8 hours. OMRON equipment (model HBF-514) will be used; the equipment is composed of a tetrapolar system with 8 electrodes that transmit an extremely low electric current of 50 kHz and 500 µA. Six variables will be recorded: basal metabolism value, body fat percentage, skeletal muscle percentage, body mass index, visceral fat percentage, and basal metabolic rate.

#### 2.6.3. Data collection related to secondary outcomes.

##### 2.6.3.1. Assessment of respiratory pressures.

Respiratory muscle strength maximum inspiratory pressure and Maximum Expiratory Pressure will be measured using an analog manovacuometer (±150 cmH_2_O; Murenas). Manovacuometry is a simple, fast, noninvasive, and voluntary test requiring participants’ collaboration and maximum effort. Participants should be seated while the researcher is in front of them providing verbal encouragement. In addition to the manovacuometer, a chair, a nose clip, a flat mouthpiece, and a trachea will be needed for performing the test. For maximum expiratory pressure, participants will be asked to inflate their lungs to total lung capacity and perform a forced expiration, sustaining the peak pressure for approximately 5 seconds. For maximum inspiratory pressure, participants will be asked to exhale up to the functional residual capacity and perform a maximal inspiration, sustaining the pressure for approximately 5 seconds.^[10]^ Participants will perform 3 acceptable and reproducible maneuvers with a maximum difference of 10% among the values.

##### 2.6.3.2. Functional autonomy.

Functional autonomy will be assessed using the Latin American Group for Maturity (GDLAM) protocol. GDLAM comprises the simulation of 5 activities of daily living, namely walking 10 m, standing up from sitting position, standing up from prone position, standing up from a chair and moving around the house, and putting on and taking off a t-shirt. The following assessment tools will be used during the protocol: a stopwatch, a tape measure, a mattress, a 50 cm chair, 2 cones, and a t-shirt.^[11]^ The time in seconds to perform each simulation will be measured, and the GDLAM index of autonomy will be calculated in scores, in which lower values indicate higher functional autonomy.

We will also conduct the Functional Reach Test. This test measures the maximum distance an individual can reach forward by leaning the trunk with arms extended and shoulder in 90° flexion, without moving heels off the ground or touching the tape. Distances smaller than 15 cm indicate fragility and risk of falls, and values equal to or smaller than 17 cm indicate a 13-fold higher risk of falls.^[12]^

##### 2.6.3.3. Cognitive function.

The Mini-Mental State Examination will assess participants’ mental state and cognitive function. It is a 30-point questionnaire grouped into 7 categories that assess specific cognitive functions, namely orientation to time (5 points), orientation to place (5 points), registration (3 points), attention and calculation (5 points), recall of 3 words (3 points), language (8 points), and visual construction (1 point). Total score ranges from 0 to 30; The maximum score is 30. A score of 23 or lower indicates cognitive impairment. The cutoff is 19 for participants with low educational level.^[13]^

##### 2.6.3.4. Balance and risk of falls.

The Time Up and Go test will assess participants’ balance, functional mobility, and risk of falls. The test asks participants to get up from an armed chair, walk 3 m, turn around, walk back to the chair, and sit down. According to the time in seconds to complete the test, participants will be classified as without risk of falls (up to 10 seconds), partial dependence and low risk of falls (11–20 seconds), and significant deficit in physical mobility and high risk of falls (over 20 seconds).^[12]^

The Berg Balance Scale will assess participants’ risk of falls and functional dependence. The test consists of 14 predetermined tasks that are common to daily living, each of which is scored on a scale from 0 to 4. The score is based on the time required to perform and maintain a position, perform a task, or reach a distance with the upper limb. The maximum score is 56; higher scores indicate better balance. Scores equal to or lower than 36 are associated with a 100% risk of fall.^[14]^

##### 2.6.3.5. Functional capacity.

The 6-minute walk test will assess participants’ functional exercise capacity and general respiratory, cardiac, and metabolic functioning. The test consists of walking at maximum and constant speed along a 30 m corridor. Cones will indicate the distance to be covered, and each meter will be marked on the floor with yellow tape. Participants will be verbally encouraged to maintain their walking speed (e.g., “You are walking very well; keep it up!” “Keep the rhythm”). The distance covered in meters will be compared with reference values for the Brazilian population.^[9]^

### 2.7. Intervention

#### 2.7.1. Exercise group.

EG will perform an exercise protocol composed of aerobic and strength exercises validated by Raimundo et al.^[8]^ The exercise protocol comprises 24 sessions of 60 minutes each and will last 12 weeks. Each session will start with a 5-minute static stretching of forearm and leg flexors and extensors, and arm abductors. Then, it will be followed by a 30-minute walk on a treadmill. During the walk, participants’ heart rate will be monitored to keep between 50% and 70% of their heart rate reserve.^[15]^ Participants will perform 20 minutes of strength exercises at 50% to 70% of their 1-repetition maximum. Strength exercises will comprise 3 sets of 10 repetitions of muscular resistance exercises for forearm and leg flexors and extensors, and arm abductors. A 5-minute deceleration exercise will be performed at the end of the session. Participants will perform 2 sets of 10 repetitions of diaphragmatic breathing in dorsal decubitus or sitting position. Vital signs (i.e., blood pressure, heart rate, respiratory rate, and oxygen saturation) will be measured before and after each session.

Training will be individual and take place at the Institute of Tisiology and Pneumology of Campina Grande, Paraíba (Dr Edgley Maciel Lacerda, CNPJ:088529880001-64, St. Fernandes Vieira, José Pinheiro, 58104-180, Campina Grande, Paraíba, Brazil), in the physical therapy service. The exercise protocol will be performed exclusively after the institutional authorization term, and a researcher with degree in physical therapy will supervise all sessions.

##### 2.7.1.1. Adherence to the exercise protocol.

Attendance to sessions will be registered, and the percentage of attendance will be calculated. The punctuality, engagement in other physical activities in addition to the protocol, number and type of adverse events, and adherence attitude during the session will also be registered. The level of perceived exertion, mood, and feeling of acute exhaustion induced by exercise will be recorded to anticipate possible fatigue symptoms or dissatisfaction and allow proper adjustments in the protocol. Adherence is essential to investigating the effectiveness of the exercise protocol; therefore, strategies will be implemented to maximize it (e.g., sending motivational messages every week and videos every month via WhatsApp).

#### 2.7.2. Control or GG.

Participants will play Just Dance (2011), a game-based therapy in which participants should follow dance movements. All movements will be tracked through the Kinect sensor to quantify performance at the end of the session. Xbox 360º device with Kinect (4 Gb; Microsoft) will be connected to a TV with compatible video formats. Participants will exercise for 60 minutes without interruption, and their heart rate will be monitored with an oximeter. Their heart rate should be between 50% and 70% of the heart rate reserve.^[15]^

### 2.8. Crossover

The first phase of the study should be carried out in 14 weeks, that is, 2 weeks of assessments (Assessment 1) and 12 weeks of intervention. After EG AND CG INTERVENTION OR INTERVENTION A, participants will be reassessed (Assessment 2). All this process may last 15 weeks. In the wash-out phase, participants will not receive any interventions for 4 weeks. Around the 20^th^ week, participants will return for assessment 3, and EG and CG will switch protocols. After the crossover phase, all participants will be reassessed (Assessment 4).

### 2.9. Follow-up

During the follow-up, all participants will receive the same treatment (i.e., regular standard care), which will allow the assessment of the medium-term effects of the exercise and gametherapy protocol.

### 2.10. Participant safety

Any adverse effects (i.e., any musculoskeletal injury that occurs during or because of the training) will be reported.

### 2.11. Participant drop out

We will record and inform the number of participants that drop out of the study and the reason.

#### 2.11.1. Participant abandonment.

Participation in this study is entirely voluntary. Participants can leave the study at any time without explaining. Leaving the study will not result in any negative consequences regarding care or treatment received.

#### 2.11.2. Interruption of the intervention by the research team.

The research team may withdraw participants at any time if their safety is at risk or the study protocol is violated, which may occur under the following circumstances:

- Severe musculoskeletal injuries that alter their usual lifestyle (whether or not as a consequence of the study participation).

- Change of residence, hindering the assessments.

- Death.

### 2.12. Statistical analysis

Baseline measures between groups will be compared. The distribution of continuous quantitative variables will be tested for normality and graphically inspected using histograms. A general linear model will analyze differences between groups for the primary and secondary outcomes. If normality assumption is not met, we will proceed with non-parametric techniques (i.e., quantile regression). Effect size (95% confidence interval) and level of statistical significance will be displayed. Missing data will be replaced using multiple imputations. We will minimize potential confounding by adjusting baseline values and controlling other potential confounders. Statistical significance will be set at *P* < .05.

### 2.13. Registration and adherence to SPIRIT standards

This study was prospectively registered in the ISRCTN registry (ISRCTN27697878) on October 4, 2019, before participant enrollment (i.e., October 15, 2019). This study will follow SPIRIT guidelines for randomized clinical trials, and results will be reported following 2010 CONSORT standards (http://www.consort-statement.org/).

## 3. Discussion

Our study will evaluate the effects of an evidence-based and supervised exercise protocol on active older adults. This study will allow translating exercise-based research into clinical practice. There is a paucity of studies regarding exercise in active older adults. Therefore, some of our study criteria will be defined based on populations other than older adults. We completed a 2-week pilot study to evaluate the exercise protocol and test its feasibility in individuals with different characteristics.

If the proposed protocol does not have the expected beneficial effect on HRV, results of this clinical trial will still be interesting to science. If we find unexpected results, this study will contribute to new ideas and research hypotheses, such as modifying the exercise protocol, combining it with other interventions (e.g., nutritional or psychological treatments), or increasing the follow-up period. We will prepare our results for submission to conferences, journal publication, and other sources without restrictions. Participants will receive personal information on their results and a summary of overall results.

### 3.1. Limitations/study risks and contingency plan

Potential risk 1: EG participants not attending to intervention sessions.

Contingency plan: Different strategies will be implemented to maximize adherence to the exercise protocol. The emotional state (The Feeling Scale) will be monitored, and any adverse events will be registered during training to understand participants’ feelings and implement adaptations. Participants will also receive motivational messages via WhatsApp every week and motivational videos from coaches and the research team at the beginning of each month.

Potential risk 2: Low recruitment and/or retention rate.

Contingency plan: The research team will invite as many active older adults to participate as possible. If the recruitment rate is still low, we will reach out to other exercise training facilities in different areas to recruit potential participants.

## Author contributions

**Conceptualization:** Sáskia Fürstenberg Thoma, Ester Laura Cordeiro Oliveira Costa, Jéssica Costa Leite, Rodrigo Daminello Raimundo.

**Data curation:** Sáskia Fürstenberg Thoma, Juliana Zangirolami-Raimundo, Rodrigo Daminello Raimundo.

**Formal analysis:** Sáskia Fürstenberg Thoma, Juliana Zangirolami-Raimundo, Rodrigo Daminello Raimundo.

**Funding acquisition:** Sáskia Fürstenberg Thoma, Rodrigo Daminello Raimundo.

**Investigation:** Sáskia Fürstenberg Thoma, Ester Laura Cordeiro Oliveira Costa, Rodrigo Daminello Raimundo.

**Methodology:** Sáskia Fürstenberg Thoma, Ester Laura Cordeiro Oliveira Costa, Airlon Nery Ferreira, Jéssica Costa Leite, Juliana Zangirolami-Raimundo, Rodrigo Daminello Raimundo.

**Project administration:** Sáskia Fürstenberg Thoma, Eduardo Antônio Costa Silva, Ester Laura Cordeiro Oliveira Costa, Airlon Nery Ferreira, Rodrigo Daminello Raimundo.

**Resources:** Sáskia Fürstenberg Thoma, Andrés Ricardo Pérez-Riera, Ester Laura Cordeiro Oliveira Costa, Airlon Nery Ferreira, Jéssica Costa Leite, Rodrigo Daminello Raimundo.

**Software:** Sáskia Fürstenberg Thoma, Rodrigo Daminello Raimundo.

**Supervision:** Sáskia Fürstenberg Thoma, Jéssica Costa Leite, Rodrigo Daminello Raimundo.

**Validation:** Sáskia Fürstenberg Thoma, Rodrigo Daminello Raimundo.

**Visualization:** Sáskia Fürstenberg Thoma, Rodrigo Daminello Raimundo.

**Writing – original draft:** Sáskia Fürstenberg Thoma, Juliana Zangirolami-Raimundo, Rodrigo Daminello Raimundo.

**Writing – review & editing:** Sáskia Fürstenberg Thoma, Emanuel Ewald Thoma Belfort, Jéssica Costa Leite, Juliana Zangirolami-Raimundo, Rodrigo Daminello Raimundo.
